# A Paradox of Fit: How Job Complexity Shapes AI Self-Efficacy and AI Adoption Through a Curvilinear Mechanism

**DOI:** 10.3390/bs15121659

**Published:** 2025-12-02

**Authors:** Mustafa Akben, Su Dong

**Affiliations:** Department of Management and Entrepreneurship, Elon University, Elon, NC 27244, USA; sdong@elon.edu

**Keywords:** job complexity, AI self-efficacy, AI adoption, Generative Artificial Intelligence

## Abstract

The rapid emergence of generative AI is transforming how employees engage with technology to perform tasks, make decisions, and create value. Despite its transformative potential, empirical findings on AI adoption remain inconsistent, particularly regarding how job characteristics shape employees’ confidence and readiness to use generative AI. Grounded in the Task–Technology Fit framework and self-efficacy theory, this research examines the curvilinear relationship between job complexity and AI self-efficacy and its subsequent effects on AI adoption readiness and behavior. We conducted two survey studies to test the proposed hypotheses using structural equation modeling. Results reveal that employees in both low- and high-complexity roles exhibit a low level of AI self-efficacy and a subsequent lower level of AI adoption behaviors compared to those in moderately complex roles. These findings challenge the assumption that highly skilled roles typically lead AI integration and instead highlight the importance of aligning task structure with AI capabilities. This study advances theory by introducing a non-linear boundary condition to technology adoption and offers practical guidance for organizations to design jobs and training programs that cultivate confidence and foster sustainable human–AI collaboration.

## 1. Introduction

Generative Artificial Intelligence (GenAI) has rapidly emerged as a transformative technology capable of reshaping how employees perform tasks, make decisions, and create value. Its potential to automate routine processes, augment complex reasoning, and accelerate knowledge work has fueled the promise of widespread organizational adoption. Yet, emerging evidence reveals that GenAI’s benefits are unevenly distributed across occupations and roles. For instance, Brynjolfsson et al. (2025) reported a 15% productivity increase among customer-support agents using AI, but these gains accrued primarily to less experienced workers, with minimal improvement observed in roles where task complexity is more prevalent. Such similar asymmetries across industries (Handa et al., 2025) indicate that the benefits of GenAI are not solely determined by individual characteristics but by the structural features of work itself. The prior literature suggests that job characteristics may influence opportunities for meaningful human–AI interaction (Przegalinska et al., 2025). Consequently, understanding the conditions under which GenAI complements or constrains employee performance requires shifting the analytical lens from merely personal adoption traits to the nature of the job as the contextual foundation for AI use. However, we still lack a clear understanding of when and how such job characteristics facilitate or constrain employees’ confidence in using GenAI and their subsequent adoption behavior.

Employees’ GenAI adoption decisions are not made in isolation. They unfold within the constraints and affordances of their work context. Job characteristics create the conditions under which individuals decide whether and how to integrate new technologies (Venkatesh et al., 2003). Among these characteristics, job complexity has become a particularly salient determinant of technology use. Job complexity reflects “the extent to which a job entails autonomy or less routine and the extent to which it allows for decision latitude” (Shalley et al., 2009, p. 493). It encompasses a range of skills required for effective task execution (Morgeson & Humphrey, 2006). This complexity might shape performance outcomes and also contribute to how employees engage with intelligent technologies (Bailey et al., 2019; Q. Zhang et al., 2025). When job complexity provides an optimal level of technological assistance, employees gain frequent, meaningful opportunities to interact with GenAI, fostering confidence and learning through positive experiences. Conversely, when job complexity is either too low or too high, opportunities for productive human–AI interaction diminish, either because work is overly routine and easily automated or because it exceeds GenAI’s current capabilities, leading to frustration and diminished confidence. These dynamics suggest a nonlinear relationship between job complexity and employees’ confidence in using AI, or AI self-efficacy (Almatrafi et al., 2024; Bandura, 1997).

These observations reveal a joint empirical and theoretical puzzle. Empirically, recent work documents uneven benefits of generative AI across jobs and that the influence of job complexity on its use is inconsistent, with studies reporting positive, negative, and curvilinear effects (e.g., Brynjolfsson et al., 2025). Theoretically, however, prevailing technology adoption models largely treat task complexity and fit as monotonic drivers of utilization and do not specify how job design shapes the development of AI self-efficacy and readiness (e.g., Awa et al., 2017). As a result, existing frameworks are muted to explain why generative AI improves performance in some roles, has little effect in others, and may even undermine confidence and use in highly complex jobs. This empirical inconsistency therefore signals a deeper theoretical gap that our study seeks to address.

**Figure 1:**
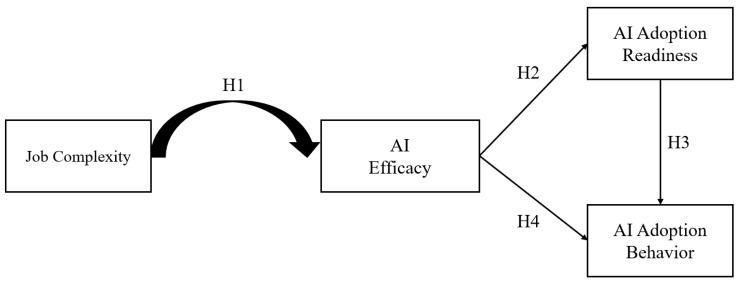
Proposed Theoretical Model.

To address this joint empirical and theoretical puzzle, this study integrates the Task–Technology Fit (TTF) framework (Goodhue & Thompson, 1995) as an overarching theoretical lens. TTF posits that technology adoption and performance depend on the alignment between task demands and technological capabilities. However, traditional TTF assumes static task–technology relationships, which may not hold for adaptive systems such as GenAI (Mollick, 2024). We extend this framework by theorizing that job complexity determines the opportunity for task–technology alignment, which is defined as GenAI’s bounded capabilities match the demands of the job. At moderate levels of complexity, this alignment facilitates frequent, successful human–AI interactions that strengthen self-efficacy and readiness for adoption. At the extremes, misfit either limits meaningful engagement or generates repeated failure experiences, undermining confidence. This reasoning suggests that job complexity exerts an inverted-U-shaped effect on AI self-efficacy, which in turn promotes readiness and actual adoption behavior. We present our proposed model in Figure 1.

To test these propositions, we conducted two empirical studies (N_1_ = 306, N_2_ = 246) in a private higher education institution in the United States, representing a context that naturally varies in job complexity and exposure to AI technologies. The multi-study design enables robust examination of the hypothesized curvilinear relationships and the mediating role of AI self-efficacy in shaping adoption outcomes.

This research advances theory and practice in three primary ways. Collectively, these contributions respond to both the empirical puzzle of uneven and inconsistent GenAI effects across jobs and the theoretical gap in how existing task–technology fit perspectives account for these patterns. First, it extends TTF theory by demonstrating that fit is not uniformly beneficial but follows a nonlinear pattern. We identify a “*paradox of fit*,” where both very low and very high job complexity undermine employees’ confidence in using AI. We also discover the inverted-U relationship that defines a “sweet spot” where task demands and AI capabilities align to foster self-efficacy. Second, the study advances the psychology of technology adoption by introducing AI self-efficacy and AI adoption readiness as key mechanisms. Rather than treating adoption as a simple outcome of intention, we show that AI adoption readiness, the active cognitive and emotional preparation to use AI, translates confidence into action. This highlights that successful adoption depends as much on psychological readiness as on technical fit. Third, the research integrates job design and technology adoption perspectives to explain how the structure of work shapes AI use. Job complexity determines whether employees encounter opportunities that build or erode confidence, positioning AI adoption as both a technical and developmental process. Practically, these findings emphasize that a one-size-fits-all approach to AI deployment is ineffective. Organizations should tailor implementation to job complexity, maintain human involvement in simpler roles, and manage expectations in more complex ones, fostering the moderate “Goldilocks zone” where human–AI collaboration is most productive.

## 2. Theory and Hypothesis

We turn to the Task Technology Fit (TTF) framework as our theoretical foundation to understand how generative AI adoption plays out. TTF theory rests on the premise that technology has a positive effect on utilization and performance when it fits with task requirements (Chung et al., 2015; Goodhue & Thompson, 1995). The antecedents of TTF include task characteristics, such as task complexity and interdependence (Campbell, 1988; Wood, 1986), and technology characteristics, such as processing speed, accuracy, and usability (Goodhue & Thompson, 1995; Zigurs & Buckland, 1998). At its core, this framework holds fit as the key to technology utilization by shaping employees’ beliefs about the technology’s usefulness and value for accomplishing their tasks. These beliefs, in turn, affect adoption decisions and ultimately impact performance (Goodhue & Thompson, 1995). In this study, we focus on the adoption pathway, examining how this alignment influences employees’ AI adoption behavior. However, applying this framework to generative AI requires careful consideration of the technology’s unique characteristics and constraints.

This consideration is particularly urgent given that empirical findings regarding the influence of job complexity on technology adoption remain inconsistent, creating mixed findings. One stream of research posits a positive linear relationship driven by rational utility, arguing that high task complexity drives the necessity for technological tools. For instance, Awa et al. (2017) suggest that employees in complex roles are more likely to adopt systems to facilitate progression, while Q. Zhang et al. (2025) argue that high complexity can act as a “challenge appraisal,” framing AI as a necessary resource that boosts self-efficacy. Conversely, a second stream suggests a negative relationship, identifying complexity as a barrier rather than a driver. Malik et al. (2022), for example, identify “techno-complexity” as a primary source of stress, arguing that when tasks are already demanding, the introduction of tools that also comes with its own complexity creates “technostress” and feelings of incompetence. This aligns with findings in healthcare contexts where high job complexity weakens the positive impact of AI because current technologies lack the reliability to handle nuanced, high-stakes ambiguity, leading to a capability mismatch (Huo et al., 2025).

Recent scholarship has attempted to resolve these contradictory findings by suggesting the relationship is curvilinear. Notably, X. Zhang et al. (2025) provide empirical evidence of an inverted U-shaped relationship in digital performance. However, their framework focuses on enterprise social media (ESM), attributing the decline at high complexity to “information overload” and social distraction. We argue that the mechanisms governing Generative AI differ fundamentally from social media. Unlike ESM, where the friction is caused by excessive input (noise), the friction in high-complexity GenAI use is caused by insufficient capability (failure). Because Generative AI possesses specific bounded capabilities, the barrier at high complexity is not that the user is overwhelmed by information, but that the AI fails to perform the necessary reasoning, leading to “enactive failure” that erodes confidence (Bandura, 2000). Thus, while we build on the curvilinear premise, we diverge from prior work by identifying AI Self-Efficacy, developed through successful mastery experiences, as the distinct psychological mechanism that explains why adoption falters at the extremes of both automation (low complexity) and capability failure (high complexity).

More specifically, applying TTF logic to generative AI (GenAI) requires recognizing that GenAI possesses bounded capabilities. GenAI excels at pattern recognition and information processing but struggles with contextual interpretation, tacit knowledge synthesis, or reasoning capabilities (Brynjolfsson et al., 2025; Jarrahi, 2018). These bounded capabilities fundamentally shape how employees interact with GenAI. When task demands align with AI’s capabilities, jobs tend to create frequent opportunities for productive human-AI collaboration that build confidence through repeated use, whereas when task demands exceed or fall short of AI’s boundaries, jobs typically provide limited meaningful opportunities for integration, constraining confidence development. This misfit between GenAI and the employee’s task is bidirectional. GenAI can misfit the user’s needs by performing the task inadequately, or it can misfit the user and task demands by exceeding the user’s needs, leading to minimal interaction with AI. We therefore extend TTF by examining how job complexity shapes interaction opportunity, the task-afforded occasions for human-AI collaboration. By aligning task demands with AI’s bounded capabilities, job complexity influences the accumulation of mastery experiences that build self-efficacy, subsequently affecting adoption decisions. In the following sections, we delineate this mechanism in detail and present the key concepts and constructs in Table 1.

### 2.1. From Task-Technology Fit to AI Self-Efficacy

TTF theory maintains that task-technology fit drives utilization through employees’ beliefs about technology usefulness and value (Goodhue & Thompson, 1995). We extend this framework by examining how fit shapes AI self-efficacy, that is, employees’ beliefs in their capability to successfully use AI to accomplish their work tasks (Compeau & Higgins, 1995). This construct differs from perceived usefulness by focusing not on whether AI is helpful, but on whether employees believe they can successfully benefit that help.

Prior technology adoption research has extensively examined self-efficacy and computer self-efficacy as a broad belief about one’s ability to use information technologies or computing systems (Compeau & Higgins, 1995; Venkatesh et al., 2003). We suggest that generative AI’s probabilistic and interactive nature may require a more nuanced conceptualization of efficacy. Recent work by Wang and Chuang (2024) demonstrates that AI self-efficacy captures AI-specific characteristics that traditional technology self-efficacy scales neglect, suggesting that the construct warrants distinct theoretical and empirical treatment. Unlike traditional systems that produce relatively predictable outputs through fixed interfaces, generative AI appears to engage users in iterative dialogue where outputs can vary in quality and accuracy, potentially requiring continuous judgment about when to trust, refine, or override algorithmic suggestions (Agrawal et al., 2019; Dell’Acqua et al., 2023; Jarrahi, 2018). Additionally, GenAI’s capabilities tend to be context-dependent and bounded, performing well at some tasks while struggling with others, which may demand that users develop discernment about when AI augments versus hinders their work. Accordingly, we conceptualize AI self-efficacy as capturing employees’ confidence in navigating these distinctive challenges. Their belief that they can effectively integrate generative AI into core work tasks through skillful prompting, critical evaluation, and sound judgment about its appropriate application. This domain-specific construct extends beyond operating AI tools to encompass the capability for productive human-AI collaboration.

We propose that job complexity, which encompasses the cognitive demands, task variety, and problem-solving requirements of work (Shalley et al., 2009; Wood, 1986), has an inverted-U relationship with AI self-efficacy. This curvilinear effect emerges through mastery experiences, which is often considered as the primary source of self-efficacy development (Bandura, 1997; Usher & Pajares, 2008). When job complexity aligns with AI’s bounded capabilities, employees accumulate positive interactions that build confidence. When it misaligns, either too low or too high, interaction opportunities diminish.

#### The Paradox of Fit: When High Technical Alignment Undermines Adoption

We define the paradox of fit as a non-monotonic pattern in which task–technology alignment that appears favorable can, under specific conditions, suppress AI self-efficacy and subsequent adoption behavior. This departs from traditional Task–Technology Fit logic, which typically assumes that closer alignment reliably improves evaluations and use (Goodhue & Thompson, 1995). In GenAI, whose value depends on iterative, human-in-the-loop interaction and mastery experiences, both “over-fit” (tasks too simple relative to capabilities) and “under-fit” (tasks exceeding capabilities) can erode confidence rather than build it (Bandura, 2000). In the following sections, we unpack how and why this pattern of relationship might emerges across varying levels of job complexity and demonstrate its implications for effective human–AI collaboration. Table 2 provides a summary of the hypotheses and their corresponding theoretical rationales.

### 2.2. The Curvilinear Effect of Job Complexity on AI Self-Efficacy

#### 2.2.1. Low Job Complexity: The Automation Paradox

At low job complexity, roles involve routine, rule-based tasks with clear procedures and predictable patterns, such as data entry, appointment scheduling, or basic document formatting. Generative AI’s capabilities substantially exceed these task demands, enabling near-complete automation with minimal human engagement (Huang & Rust, 2018). This creates a paradox that challenges TTF predictions. While AI fits these tasks technically, automation eliminates the human-AI interactions necessary for self-efficacy development. Without ongoing interaction opportunities, employees cannot accumulate the mastery experiences that build confidence. Consider an administrative assistant whose scheduling is handled by Gen AI. After configuring initial preferences and rules, they rarely engage with the system as it autonomously manages appointments, sends reminders, and resolves conflicts. Such employees have few opportunities to develop and demonstrate their capability to leverage Gen AI effectively. Thus, despite high technical fit, AI self-efficacy might remain underdeveloped at low job complexity. This represents a situation where this high degree of fit, driven by GenAI’s excessive capacity, inadvertently worsens human-AI collaboration opportunities.

#### 2.2.2. Moderate Job Complexity: The Augmentation Sweet Spot

At moderate job complexity, roles, such as market analysis, report synthesis, and strategic recommendations, require judgment and contextual understanding that prevent full automation (Marler, 2024; Raisch & Krakowski, 2021). These requirements create the optimal conditions for human-AI collaboration. Generative AI handles information processing and routine analysis while humans provide quality assessment, ethical considerations, and expert reasoning, establishing a “human-in-the-loop” dynamic with frequent interaction opportunities (Jarrahi, 2018). Such interactions constitute guided mastery experiences where AI scaffolds performance while employees retain decision-making control. These are the ideal conditions for self-efficacy development (Bandura, 1997). Consider how a market analyst uses AI to process survey data and identify patterns while applying human judgment to interpret strategic implications. Each task requires interdependent collaboration that reinforces the employee’s capability. This continuous stream of successful interactions enables employees to build evidence of their ability to leverage AI effectively and thus contribute their belief in their confidence building (Dell’Acqua et al., 2023). Unlike the automation paradox at low complexity, moderate complexity maximizes self-efficacy development through sustained, meaningful human-AI collaboration opportunities.

#### 2.2.3. High Job Complexity: The Capability Ceiling

At high job complexity, roles involve strategic decisions, stakeholder negotiations, and policy development that demand tacit knowledge, political navigation, and judgment based on years of institutional expertise (Shalley et al., 2009; Simon, 1991). These core requirements exceed generative AI’s current capabilities in contextual interpretation and nuanced reasoning, creating a fundamental task-technology misfit (Goodhue & Thompson, 1995). This misfit becomes evident in practice. Consider a hospital executive developing strategic responses to new regulatory changes. She inputs institutional context, past policies, and stakeholder concerns, expecting nuanced recommendations. Instead, AI generates generic strategies that overlook organizational constraints, misread political dynamics, and ignore the institutional knowledge essential for implementation (Lebovitz et al., 2021). After attempts yield similar failures, employees in high-complexity roles might accurately assess that AI cannot handle their core strategic work yet. These negative mastery experiences reduce employees’ willingness to interact with and explore AI’s capabilities, creating a self-reinforcing cycle where limited interaction hinders the learning and navigation needed to develop further confidence in using AI (Venkatesh, 2000). Thus, at high complexity, evidence-based assessment of poor task-technology fit leads to diminished self-efficacy. Taken together, we hypothesized that:

**H1.**  *Job complexity has an inverted-U shaped relationship with AI self-efficacy, such that AI self-efficacy initially increases as job complexity moves from low to moderate levels but decreases as job complexity increases beyond moderate levels to high complexity.*

### 2.3. AI Self-Efficacy and Adoption Readiness

We propose that AI self-efficacy positively influences AI adoption readiness, the extent to which individuals prepare to integrate AI beyond minimal task requirements (Armenakis et al., 1993; Blut & Wang, 2020; Rafferty et al., 2013). This readiness represents a multi-dimensional construct encompassing cognitive understanding of AI’s capabilities and boundaries, affective orientation toward AI integration, and forming concrete implementation intentions (Armenakis et al., 1993; Rafferty et al., 2013). Developing these dimensions requires allocating limited cognitive and emotional resources, with self-efficacy influencing whether employees make this investment (Bandura, 1997). More specifically, employees with high self-efficacy, having accumulated positive mastery experiences, allocate resources across all three dimensions. They invest in learning AI’s capabilities and experimenting with applications (cognitive), develop positive orientations toward integration (affective), and form concrete implementation plans (intentional). This allocation pattern reflects self-efficacy’s motivational function. Individuals with high self-efficacy expect returns from preparation efforts and thus invest in skill development (Bandura, 1997; Colquitt et al., 2000). Preparation proves especially critical for generative AI because successful adoption requires understanding the technology’s boundaries and developing judgment capabilities for output evaluation. These capabilities emerge through deliberate practice rather than intuitive use. In line with this reasoning, empirical evidence also supports this pathway in both technology adoption (Parasuraman, 2000) and organizational change contexts (Cunningham et al., 2002). Thus, we hypothesize that:

**H2.**  *AI self-efficacy is positively related to AI adoption readiness.*

### 2.4. AI Adoption Readiness and AI Adoption Behavior

We propose that AI adoption readiness positively influences AI adoption behavior, which we define as the frequency and extent of actual AI use in daily work. Self-efficacy captures capability beliefs developed through mastery experiences, while readiness captures preparedness built through deliberate skill investment (Armenakis et al., 1993). This distinction matters because capability beliefs require practical implementation skills to translate into action (Ajzen, 1991; Venkatesh et al., 2003). Technology adoption research documented that facilitating conditions such as skills, knowledge, and practical understanding influence whether positive beliefs translate into usage (Venkatesh et al., 2003). We argue that readiness provides these facilitating conditions by equipping employees with procedural and practical knowledge for AI integration. Those who invest in readiness develop practical knowledge of when and how AI aligns with task demands and act decisively, while those without such preparation hesitate the act and adopt the new technology in daily uses (Venkatesh et al., 2003). Research on organizational change consistently shows that readiness, not merely positive attitudes, strongly predict whether employees successfully adopt new technologies (Holt et al., 2007; Weiner, 2009). Even motivated employees struggle to translate intentions into action without the concrete skills, practical knowledge that readiness provides (Taylor & Todd, 1995; Venkatesh et al., 2003). Thus, we hypothesize that

**H3.**  *AI adoption readiness is positively related to AI adoption behavior.*

### 2.5. AI Self-Efficacy as a Direct Driver of AI Adoption

We argue that AI self-efficacy directly influences AI adoption behavior beyond its indirect effect through readiness. This direct path matters because generative AI’s context-dependent and probabilistic nature requires iterative refinement (Knoth et al., 2024). Outputs vary with each use, demanding persistence through trial-and-error (Agrawal et al., 2019). Self-efficacy shapes willingness to voluntarily expand AI use beyond requirements and influences persistence through these inevitable variations (Bandura, 1997). When encountering unhelpful AI outputs, employees with low self-efficacy interpret failures as confirmation of unsuitability and abandon use, while those with high self-efficacy view failures as surmountable challenges and persist through iteration (Han et al., 2025). Self-efficacy operates independently of readiness. Even employees with practical capabilities need confidence to persist through such challenges rather than abandoning attempts prematurely (Ajzen, 1991; Marakas et al., 1998). Empirical evidence confirms this direct path. self-efficacy predicts technology persistence and continued usage beyond initial adoption, determines acceptance or rejection of AI-generated suggestions, and consistently influences adoption behavior across information systems contexts (Compeau & Higgins, 1995; Dietvorst et al., 2015; Hong et al., 2002; Venkatesh et al., 2003). Thus, we hypothesize that:

**H4.**  *AI self-efficacy is positively related to AI adoption behavior.*

## 3. Method

We conducted two cross-sectional survey studies to examine the relationships between job complexity, AI self-efficacy, and AI adoption. Both studies employed self-report measures from independent samples. The studies were approved by the University Institutional Review Board with the protocol #23-2769. Prior to presenting the AI-related measures, participants were provided with a brief definition of generative AI and large language models to ensure common understanding across respondents with varying levels of technical expertise. During the preparation of this manuscript, the authors used ChatGPT 5.1 to assist with language refinement and clarity of expression such as grammar corrections. The authors carefully reviewed and edited all generated text and take full responsibility for the final content of this publication.

### 3.1. Study 1

#### 3.1.1. Study 1 Participants and Procedure

In Study 1, we collected data from an initial pool of 402 respondents, yielding 306 complete responses for analysis with a completion rate of 76.1%. The sample comprised faculty members (46%), staff members (47.7%), and individuals serving in both roles (6.3%). Participants were predominantly women (55.9%) and White (80.4%), with a mean organizational tenure of 9.26 years (SD = 8.32) and average work experience of 19.38 years (SD = 9.93). Data were collected from a mid-sized private university in the southeastern United States during summer 2023. Participants were recruited through campus-wide emails distributed to all full-time faculty and staff members aged 18 years and older. Informed consent was obtained before the survey began. The survey was administered via the Qualtrics platform. Participation was voluntary.

#### 3.1.2. Study 1 Measures

Unless otherwise noted, items used a 5-point Likert scale (1 = strongly disagree, 5 = strongly agree).

**Job Complexity.** In Study 1, we assessed job complexity through a systematic coding procedure based on O*NET (Occupational Information Network) definitions. We operationalized job complexity using the O*NET classification system, following the guide by Peterson et al. (2001). Two trained coders independently evaluated each of the distinct roles that survey respondents provided in our sample (e.g., Director of Alumni Engagement, Event Coordinator, Marketing Specialist, or Dean of School, and so on), rating job complexity on a scale from 1 (low complexity) to 5 (high complexity). Specifically, coders evaluated each role along four established dimensions from O*NET content and job-design research such as task variety (the range and diversity of activities), cognitive demands (the level of complex problem-solving and critical thinking required), problem-solving requirements (the extent of non-routine or ambiguous problems encountered), and decision-making autonomy (the degree of discretion and independence in daily decisions). (Morgeson & Humphrey, 2006; Wood, 1986). Coders integrated these four dimensions into a single holistic complexity rating using behavioral anchors provided in a standardized codebook. Inter-rater reliability was strong (r = 0.95), and discrepancies were resolved through discussion in which coders revisited the original role descriptions, compared each rating against the codebook’s defined anchors, and collaboratively reached consensus. Initial differences of one or more points triggered these consensus discussions, and the resolution process continued until 100% agreement was achieved on all final complexity ratings.

**AI Self-Efficacy.** We measured AI self-efficacy using items adapted from established computer self-efficacy measures (Compeau & Higgins, 1995). We selected three items to capture confidence in integrating AI technologies into daily work. An example item is, “I feel confident in using AI in my daily work.” Cronbach’s *α* = 0.90.

**AI Readiness.** We measured AI adoption readiness following established technology adoption frameworks (Venkatesh et al., 2003) with three items assessing participants’ readiness to use AI. Participants rated their agreement with the following statements: “I intend to use AI and language models to improve my work,” “I am planning to use AI in my daily tasks,” and “I am preparing to incorporate AI in my workflow.” Cronbach’s *α* = 0.90.

**AI Adoption Behavior.** We assessed AI adoption behavior frequency using items adapted from Venkatesh et al. (2003) behavioral measure. Participants reported how often they use AI in their daily work on a 5-point scale (1 = Never, 5 = Always) across three items: “How frequently do you interact with generative AI technologies, such as ChatGPT, in your daily work?”, “How often do you find yourself using AI technologies for professional tasks or assignments?”, and “To what extent do AI technologies factor into your everyday activities at work?” Cronbach’s *α* = 0.91.

**Control Variables.** We control for the breadth of participants’ AI learning experiences by asking them to indicate all the sources through which they had learned about AI technologies. Participants were presented with a list of learning sources including, (a) external workshops or training sessions, (b) internal workshops organized by their institution, (c) self-directed learning through reading or online resources, and (d) other sources. An additional option of “none” was provided for those who had not engaged in any activities to learn about AI. The learning sources variable was computed as a count of the number of different sources selected (range 0–4), capturing the diversity of participants’ learning approaches rather than any single learning modality. This approach is consistent with research demonstrating that engagement with multiple learning sources enhances professional development and technology literacy, as diverse learning activities complement each other and are particularly important for developing complex competencies in rapidly evolving technological domains (Goller, 2017; Hornberger et al., 2023).

We included several other control variables to isolate the effects of job complexity on AI outcomes. Age was measured using ordinal categories representing age ranges (1 = 18–25 years, 2 = 26–30 years, 3 = 31–35 years, and so forth) to control for generational differences that have been shown to influence technology adoption patterns, with younger workers typically demonstrating greater comfort and facility with new technologies (Venkatesh et al., 2003). Race was coded as a binary variable (0 = non-White, 1 = White) to control for potential disparities in technology access and digital literacy that have been documented across racial groups (Van Dijk, 2020). Academic role was coded as a binary variable (0 = non-faculty, 1 = faculty) because these groups may face different technological demands and have varying levels of autonomy in choosing whether to adopt new tools. Finally, organizational tenure was measured as years at the institution and was included as longer-tenured employees may have more established work routines that could influence their openness to adopting new technologies (Agarwal & Prasad, 1999). These control variables help ensure that any observed relationships between job complexity and AI outcomes are not confounded by demographic or positional factors.

#### 3.1.3. Study 1 Analytic Strategy

All analyses were conducted using R (version 4.3.2) and the lavaan package (version 0.6-19) (Rosseel, 2012) for structural equation modeling (SEM). To test the hypothesized inverted-U relationship, path analysis with moderated mediation was employed (Preacher et al., 2007). Job complexity was mean-centered prior to creating the quadratic term, reducing multicollinearity and facilitating interpretation of interaction effects (Aiken et al., 1991). Bootstrap confidence intervals based on 10,000 resamples were used to assess the significance of indirect effects, as bootstrapping does not assume normality of the sampling distribution (Preacher & Hayes, 2008). Conditional indirect effects were examined at low (−1 SD), mean, and high (+1 SD) levels of job complexity. The model specification included direct paths from both linear and quadratic terms of job complexity to AI efficacy, serial mediation pathways through AI efficacy and AI readiness to AI adoption behavior, and direct effects from AI efficacy to AI adoption behavior. All control variables were included in each regression equation to isolate the effects of theoretical interest.

#### 3.1.4. Study 1 Results

Table 3 shows the descriptive statistics, including means, standard deviations, and zero-order correlations for all study variables in Study 1.

**Confirmatory Factor Analysis**. To evaluate the validity of the measurement model, confirmatory factor analyses (CFA) were conducted. Table 4 shows the detailed results. We compared our hypothesized measurement model, which included the three distinct latent factors (AI Efficacy, AI Readiness, and AI Adoption Behavior), against alternative, more constrained models. In this analysis, the observed Job Complexity variable was included to test its distinction from the latent constructs. The hypothesized three-factor model (with Job Complexity included as an observed variable) demonstrated good fit in Study 1 (χ^2^(30) = 100.72, CFI = 0.97, TLI = 0.96, RMSEA = 0.087, SRMR = 0.038) and was superior to alternative models. The three-factor model showed poor fit (Study 1: CFI = 0.85, RMSEA = 0.182), as did the one-factor model (in which all AI-related items were combined) (Study 1: CFI = 0.73, RMSEA = 0.243). Chi-square difference tests confirmed that the hypothesized model fit significantly better than alternative models.

**Convergent and Discriminant Validity.** We tested convergent and discriminant validity using established criteria (Fornell & Larcker, 1981; Hair et al., 2010). Table 5 shows the results. For convergent validity, we examined composite reliability (CR) and average variance extracted (AVE). All constructs exceeded the recommended thresholds, with composite reliabilities ranging from 0.90 to 0.95 (threshold: CR > 0.70) and AVE values ranging from 0.74 to 0.87 (threshold: AVE > 0.50). For discriminant validity, we applied the Fornell-Larcker criterion and verified that the square root of AVE for each construct exceeded all inter-construct correlations. We also confirmed that maximum shared variance and average shared variance remained below AVE for all constructs. These results establish both convergent and discriminant validity across both studies.

**Common Method Biases.** We assessed potential common method bias using the common latent factor (CLF) approach (Podsakoff et al., 2003). We observed a possible indication of common method variance in Study 1. To assess whether it may have an effect on the hypothesized path analytical model, we compared models with and without an unmeasured latent method factor to determine whether common method variance substantially changed the results. The findings showed no significant differences between models, indicating that common method bias was not a significant threat to the validity of the findings. Detailed results of this analysis are available upon request.

#### 3.1.5. Study 1 Hypothesis Testing

Table 6 shows the path-analytic results for Study 1.

**Figure 2:**
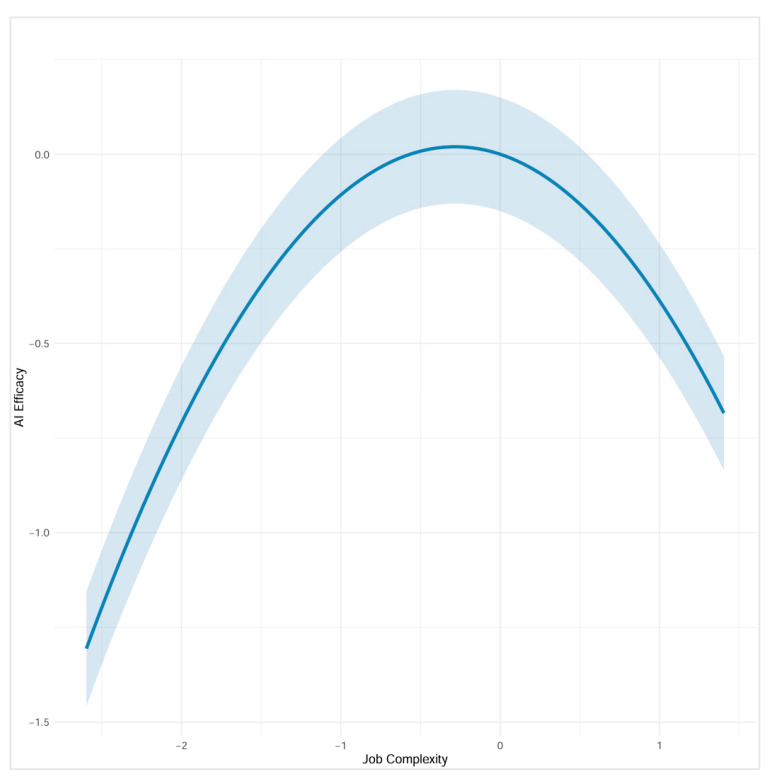
The Inverted-U Relationship Between Job Complexity (Objective ONET-Based Measure) and AI Self-Efficacy (Study 1).

Hypothesis 1 proposed that job complexity has an inverted-U shaped relationship with AI self-efficacy, such that AI self-efficacy initially increases with job complexity but decreases beyond an optimal point. We tested this hypothesis by examining both the linear and quadratic effects of job complexity on AI self-efficacy. The linear effect of job complexity on AI self-efficacy was not significant (B = −0.108, SE = 0.089, *β* = −0.076, *p* = 0.223), but the quadratic effect was significant and negative (B = −0.205, SE = 0.075, *β* = −0.167, *p* = 0.006). To interpret the curvilinear relationship, we examined simple slopes at low (−1 SD), mean, and high (+1 SD) levels of job complexity. The slope of job complexity on AI self-efficacy was positive but non-significant at low complexity (slope = 0.163, SE = 0.097, *p* = 0.094), non-significant at mean complexity (slope = −0.108, SE = 0.089, *p* = 0.223), and significantly negative at high complexity (slope = −0.379, SE = 0.163, *p* = 0.020). Figure 2 displays the curvilinear relationship between job complexity and AI self-efficacy for Study 1. These patterns demonstrate an inverted-U relationship, where AI self-efficacy increases at lower levels of job complexity but decreases at higher levels of complexity. These findings support Hypothesis 1.

Hypothesis 2 proposed that AI self-efficacy is positively related to AI adoption readiness. AI self-efficacy strongly predicted AI adoption readiness (B = 0.712, SE = 0.063, *β* = 0.699, *p* < 0.001). These results indicate that individuals with higher confidence in using AI technologies showed greater readiness to adopt AI in their work. These findings support Hypothesis 2.

Hypothesis 3 proposed that AI readiness is positively related to AI adoption behavior. AI adoption readiness significantly predicted AI adoption behavior (B = 0.497, SE = 0.073, *β* = 0.537, *p* < 0.001). These results indicate that individuals who were more prepared and willing to adopt AI demonstrated higher actual AI adoption behavior in their daily work. These findings support Hypothesis 3.

Hypothesis 4 proposed that AI self-efficacy is positively related to AI adoption behavior. AI self-efficacy significantly predicted AI adoption behavior (B = 0.198, SE = 0.078, *β* = 0.211, *p* = 0.011). These results indicate that individuals with higher AI self-efficacy demonstrated greater frequency of AI adoption behavior. These findings support Hypothesis 4.

#### 3.1.6. Study 1 Indirect Effects

Although we did not hypothesize this explicitly, we tested the indirect effects to investigate how job complexity might have an indirect effect on AI adoption behavior through the serial mediation pathway (job complexity → AI self-efficacy → AI readiness → AI adoption behavior). The quadratic indirect effect through AI self-efficacy and AI readiness to AI adoption behavior was also significant (B = −0.073, 95% CI [−0.130, −0.015], *p* = 0.012). These results indicate that the inverted-U relationship between job complexity and AI self-efficacy carries through to influence AI adoption behavior via the serial mediation pathway. We further examined conditional indirect effects at different levels of job complexity. At low levels of job complexity (−1 SD), the indirect effect to AI adoption behavior was positive but not significant (B = 0.090, 95% CI [−0.028, 0.191]). At mean levels of job complexity, the indirect effect was negative but not significant (B = −0.060, 95% CI [−0.158, 0.037]). At high levels of job complexity (+1 SD), the indirect effect was negative and significant (B = −0.209, 95% CI [−0.394, −0.024]). These patterns demonstrate that at higher levels of job complexity, the negative effect on AI self-efficacy translates into reduced AI adoption behavior through decreased AI readiness, as expected based on our models.

#### 3.1.7. Summary of Study 1

Overall, the results from Study 1 find initial evidence to support all four hypotheses, establishing a curvilinear relationship. Furthermore, we observed a serially mediated relationship between job complexity and AI adoption. However, we operationalized job complexity for this study using an objective coding system based on O*NET classifications of participant job titles. Recognizing that an individual’s perception of their work’s complexity is also a critical factor, and they may provide a much more nuanced interpretation of their job complexity, we designed a second study to replicate these findings using a validated, self-report job complexity measure to ensure our model holds from both an objective and subjective standpoints.

### 3.2. Study 2

#### 3.2.1. Study 2 Participants and Procedure

The data were collected during summer 2025 using the identical recruitment and survey procedure as Study 1. Study 2 yielded 246 complete responses from 319 initial respondents with a completion rate of 77.1%. This sample included faculty members (53.3%), staff members (38.2%), and individuals in dual roles (8.5%). Participants were predominantly women (54.5%) and White (77.2%), with a mean organizational tenure of 8.92 years (SD = 8.11) and average work experience of 20.09 years (SD = 9.50). In Study 2, we replicated the design of Study 1 with different participants and a different point in time, specifically aiming to test our hypotheses with a psychometrically validated measure of job complexity.

#### 3.2.2. Study 2 Measures

We used the exact same measure for all constructs except for job complexity. The Cronbach’s *alpha* (*α*) for the replicated measures were as follows: AI Self-Efficacy (0.93), AI Readiness (0.95), and AI Adoption Behavior (0.95).

**Job Complexity.** In Study 2, job complexity was measured using a 3-item scale adapted from Zacher et al. (2010). Participants rated their agreement with statements assessing the complexity of their work tasks. A sample item is “My current work tasks are very complex.” For job complexity in Study 2, Cronbach’s *α* = 0.89.

#### 3.2.3. Study 2 Analytic Strategy

We employed the same analytic strategy as in Study 1.

#### 3.2.4. Study 2 Results

Descriptive statistics and correlations for Study 2 are presented in Table 7.

**Confirmatory Factor Analysis.** Table 8 presents the confirmatory factor analysis results for Study 2. The hypothesized four-factor model demonstrated good fit in Study 2 (*χ*^2^(48) = 107.90, CFI = 0.98, TLI = 0.97, RMSEA = 0.070, SRMR = 0.044) and was superior to alternative models. The three-factor model showed poor fit (Study 2: CFI = 0.95, RMSEA = 0.116), as did the two-factor model (Study 2: CFI = 0.84, RMSEA = 0.194). Chi-square difference tests confirmed that the hypothesized model fit significantly better than alternative models.

**Convergent and Discriminant Validity.** Similar to Study 1, we tested convergent and discriminant validity using established criteria. Table 9 presents the results. For convergent validity, we examined composite reliability (CR) and average variance extracted (AVE). All constructs exceeded the recommended thresholds, with composite reliabilities ranging from 0.90 to 0.95 (threshold: CR > 0.70) and AVE values ranging from 0.74 to 0.87 (threshold: AVE > 0.50). Consistent with Study 1, these results establish both convergent and discriminant validity.

**Common Method Biases.** We again tested potential common method bias using the common latent factor (CLF) approach. We compared models with and without an unmeasured latent method factor to determine whether common method variance substantially affected the results. The findings showed no significant differences between models, indicating that common method bias was not a significant threat to validity. Detailed results of this analysis are available upon request.

#### 3.2.5. Study 2 Hypothesis Testing

Table 10 presents the path analysis results for Study 2.

**Figure 3:**
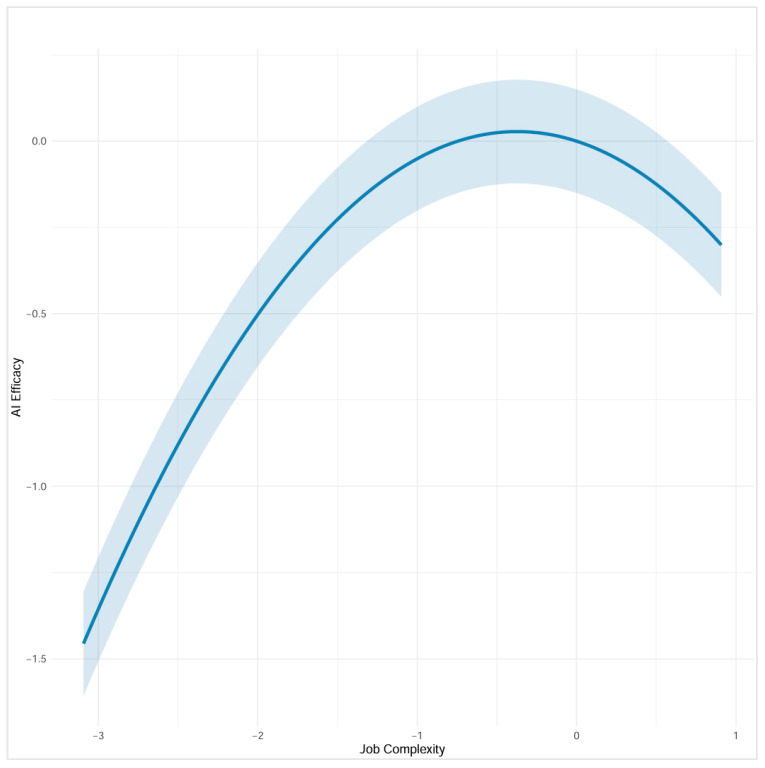
The Inverted-U Relationship Between Job Complexity (Self-rated) and AI Self-Efficacy (Study 2).

In Study 2, we replicated the findings from Study 1, with a non-significant linear effect (B = −0.152, SE = 0.115, *β* = −0.100, *p* = 0.186) and a significant negative quadratic effect (B = −0.204, SE = 0.089, *β* = −0.175, *p* = 0.022). Similarly, in Study 2, the slope was positive but non-significant at low complexity (slope = 0.187, SE = 0.129, *p* = 0.146), non-significant at mean complexity (slope = −0.152, SE = 0.115, *p* = 0.184), and significantly negative at high complexity (slope = −0.492, SE = 0.228, *p* = 0.031). Figure 3 displays the curvilinear relationship between job complexity and AI self-efficacy for Study 2. These patterns demonstrate an inverted-U relationship, where AI self-efficacy increases at lower levels of job complexity but decreases at higher levels of complexity, consistent with the proposed Hypothesis 1.

We replicated the strong relationship from Study 1, with AI self-efficacy significantly predicting AI readiness (B = 0.846, SE = 0.041, *β* = 0.794, *p* < 0.001). These results indicate that individuals with higher confidence in using AI technologies showed greater readiness to adopt AI in their work. These findings support Hypothesis 2 across both studies.

We also replicated the finding from Study 1, with AI readiness significantly predicting AI adoption behavior (B = 0.340, SE = 0.071, *β* = 0.424, *p* < 0.001). These results indicate that individuals who were more prepared and willing to adopt AI demonstrated higher actual AI adoption behavior in their daily work, consistent with Hypothesis 3.

Finaly, we replicated the finding from Study 1, with AI self-efficacy significantly predicting AI adoption behavior (B = 0.344, SE = 0.071, *β* = 0.404, *p* < 0.001). These results indicate that individuals with higher AI self-efficacy demonstrated greater frequency of AI adoption behavior in their daily work. These findings support Hypothesis 4 across both studies.

#### 3.2.6. Study 2 Indirect Effects

Similar to Study 1, we also tested possible indirect serial mediation from job complexity to AI adoption behavior through AI self-efficacy and AI adoption readiness. We again replicated the finding from Study 1 (B = −0.058, 95% CI [−0.124, −0.010], *p* = 0.043). At low levels of job complexity (−1 SD), the indirect effect on AI adoption behavior was positive but not significant in Study 2 (B = 0.118, 95% CI [−0.041, 0.278]). At mean levels of job complexity, the indirect effect was negative but not significant in Study 2 (B = −0.096, 95% CI [−0.244, 0.046]). At high levels of job complexity (+1 SD), the indirect effect was negative and significant in Study 2 (B = −0.311, 95% CI [−0.608, −0.036]). These patterns demonstrate that at higher levels of job complexity, the negative effect on AI self-efficacy translates into reduced AI adoption behavior through decreased AI readiness.

#### 3.2.7. Summary of Study 2

The results of Study 2 replicated the full pattern of findings from Study 1 with an independent sample surveyed at a different time point. We again found support for the inverted-U relationship and the full serial mediation model. Crucially, these findings were replicated using a validated, self-report measure of job complexity, demonstrating that the observed relationships are robust regardless of whether complexity is measured through objective or subjective employee perceptions.

## 4. Discussion

In this research, we have proposed and found support for a non-linear relationship between job complexity and AI adoption. Our psychological pathway model offers a critical extension to Task-Technology Fit (TTF) theory, revealing why and how employees’ psychological responses to generative AI are shaped by their work structure. We found a “paradox of fit” that manifests as an inverted-U relationship. At low job complexity, we documented that high technical fit paradoxically undermines the human-AI interactions necessary for AI self-efficacy development, while at high job complexity, a clear capability misfit also undermines its utility. These findings make distinct contributions to our understanding of Task-Technology Fit, the formation of self-efficacy in human-AI collaboration, and the nature of technology adoption pathways, which we elaborate on below.

### 4.1. Theoretical Implications

Our findings offer a contribution to Task-Technology Fit (TTF) theory, particularly in its application to generative AI. In much of the technology adoption literature, “fit” is cast as a simple, linear good, where a better technical match invariably leads to more positive outcomes like adoption and performance. Our findings challenge this linear fit assumption, suggesting instead that “fit” can be a non-linear, inverted-U relationship. While the negative effect of excessive job complexity is clear, the positive slope from low to moderate complexity is modest in magnitude. We cautiously interpret this pattern, emphasizing its theoretical coherence and moderate practical significance over its statistical strength. Our empirical results are consistent with conceptual work on human-AI collaboration, which has long argued for an optimal “augmentation” partnership, distinct from full automation on one side and capability failure on the other. Our research adds critical and empirical nuance to this idea and extends it in two crucial ways. First, we provide replicated evidence for this non-linear curve at different times and by using different measures of job complexity. Second, and more importantly, we extend this reasoning by identifying the psychological mechanism, which is AI self-efficacy developed through interaction opportunities, that explains why this “Goldilocks zone” of augmentation is so critical for adoption and development of AI self-efficacy, where individuals feel confident in using AI in their task, thus leading to daily use and much greater adoption later on through their interactions.

Our research also reveals a surprising paradox for organizations and their most high-complexity roles. Scholars have suggested that the greatest strategic value of generative AI lies in its ability to augment these critical, non-routine roles where job complexity may be high (Qian & Xie, 2025). This is precisely where human-AI collaboration is seen as most vital for innovation and strategic decision-making. We found that their AI self-efficacy significantly declines under these very circumstances, as employees in high-complexity jobs might not have opportunity to engage AI for their core strategic tasks. This evidence suggests that these employees pay a high price for their attempts at augmentation. They experience enactive failure as the AI’s current capabilities are exceeded, leading to frustration, tool abandonment, and the erosion of their confidence in the technology. These findings both challenge and qualify the overly simplistic proposition that generative AI is a universal augmentation tool. Rather, our findings indicate that without careful task alignment, attempts at high-level augmentation can backfire, eroding the very confidence organizations hope to build in their most critical employees.

Finally, our research offers a novel contribution to the technology adoption literature. Although organizational scholars have devoted a great deal of attention to the role of behavioral intentions in driving technology use, they have largely neglected the deeper, more active psychological state of adoption readiness. The limited body of research on this topic has yielded mixed, inconsistent results, with scholars frequently noting a persistent “intention-behavior gap” where positive intentions fail to translate into actual use (Jeyaraj et al., 2023). Our research takes a step toward adding new insights by introducing AI Adoption Readiness as a critical intervening variable. We show that for complex and interactive technologies like generative AI, a simple “intention to use” is insufficient. Our findings suggest that “readiness”, a richer construct capturing active cognitive and affective preparation, is the true psychological bridge that translates belief (efficacy) into action (use). These findings suggest that to truly understand when and how positive beliefs shape behavior, researchers need to consider the pivotal role of psychological readiness, rather than relying solely on the more tenuous measure of behavioral intention.

### 4.2. Practical Implications

Our findings offer clear practical guidance for leaders and managers navigating the implementation of generative AI. The observed inverted-U relationship, though moderate in effect size, suggests that a one-size-fits-all deployment strategy is unlikely to succeed. The design of the job itself remains a critical determinant of AI adoption outcomes, and organizations should interpret these effects as indicative rather than definitive. For instance, in low-complexity roles, managers must be wary of the automation paradox. While it may be efficient to fully automate routine tasks, our findings imply this robs employees of the very interaction opportunities needed to build AI self-efficacy. A more prudent strategy would involve redesigning these roles to maintain a human-in-the-loop, thereby building the confidence necessary for future, more advanced AI adoption. Conversely, in high-complexity roles, our results suggest managers must proactively manage expectations to avoid the capability ceiling. Deploying AI as a supposed strategic partner in these roles may lead to repeated enactive failure experiences that erode efficacy and build resistance. Instead, AI should be framed as a specialized assistant for discrete, well-defined sub-tasks, such as information synthesis or drafting initial communications, rather than a solution for core strategic problems. This approach properly identifies moderately complex roles as the sweet spot for augmentation, where human-AI collaboration can be most productively fostered. These insights directly address the inconsistency in prior research noted in the introduction, where empirical findings on AI adoption varied widely across occupations and levels of job complexity.

Our results also carry significant implications for employee and organizational development, which address the research gap identified in the introduction regarding the limited understanding of psychological mechanisms in AI adoption. Our psychological pathway model reveals that AI adoption is not just a technical problem, but also one of psychological development. For organizations and HR leaders, this means training must evolve beyond teaching technical skills. Our findings indicate that AI Adoption Readiness is a critical bridge between confidence and use. Therefore, training interventions must be explicitly designed to build judgment. This includes teaching employees when to use AI, how to critically evaluate its probabilistic outputs, and what its true limitations are. For employees, our model highlights that AI self-efficacy is the engine that drives this entire process. Rather than waiting for formal training, employees can take an active role by proactively seeking out guided mastery experiences, which are small, achievable, confidence boosting wins with AI. Using the tool for a discrete sub-task they know it can handle provides the initial, successful interaction that is the necessary fuel for the harder work of building deep readiness.

### 4.3. Limitations and Future Directions

The contributions of this research should be contextualized within a few key boundaries, which in turn highlight promising avenues for future inquiry. First, our study’s cross-sectional design limits our ability to make definitive causal inferences about the psychological pathway we proposed. It is certainly possible that the relationships are reciprocal. For example, AI self-efficacy may not only lead to AI use, but successful use may also build efficacy, creating a positive reinforcement loop. On the other hand, it is also possible that employees high in readiness are the ones who proactively seek out the very AI interactions that build their self-efficacy in the first place. The cross-sectional design may also limit our ability to confirm the curvilinear nature of the job complexity–AI self-efficacy relationship. Future longitudinal or experimental research could better determine whether changes in job complexity over time predict corresponding non-linear changes in AI self-efficacy, strengthening evidence for the proposed inverted-U pattern. While longitudinal models are a critical next step, establishing the fundamental associative structure of this pathway is a necessary and non-trivial first step. Our research is the first to provide robust, replicated evidence that this specific non-linear relationship exists and is associated with this psychological mechanism. Without this preliminary cross-sectional evidence, any causal investigation would be premature. Nevertheless, future research should build directly on our findings by using longitudinal, cross-lagged panel designs to untangle this co-evolutionary relationship between efficacy, readiness, and use over time.

Our findings also point to several more technical limitations that offer a clear agenda for future work. The study’s single-institution context constrains generalizability, and the O*NET-based job complexity measure in Study 1 provides only a simplified representation of work structure. While this setting allowed us to test our model within a consistent organizational culture and resource environment, it also represents a boundary condition. Faculty and staff in higher education face unique job demands, levels of autonomy, and institutional pressures regarding technology adoption that may differ significantly from those in corporate, government, or non-profit sectors. The inverted-U relationship we observed might shift or change shape in environments with different cultural norms, resource constraints, or performance incentives. Therefore, the proposed “paradox of fit” may not manifest uniformly across all contexts. For example, organizations mandate collaborative AI workflows with user training programs may experience limited curvilinear effects. Identifying such boundary conditions will clarify when and why the paradox of fit emerges, or fails to appear. Future research should therefore seek to replicate these findings across diverse industries and organizational contexts to establish the broader generalizability of our psychological pathway model. Furthermore, our dependent variable, AI adoption, was measured as frequency. This measure, however, is a crude proxy for a complex behavior. It is possible that the pathway we identified predicts frequent use, but not necessarily effective or appropriate use. For example, the quality of interactions, the amount of time spent, and adoption across different domains and use cases might be possible investigation areas. Additionally, AI adoption readiness and behavioral intention share conceptual proximity. Future research could empirically differentiate them using longitudinal or experimental designs. Finally, our model theorizes the specific psychological mechanisms linking job complexity to self-efficacy, such as enactive failures at the high end, but we did not directly measure these mediating processes. Future research might also want to investigate the exact mechanism that we describe, such as how mastery experiences are gained at different job levels and how this process directly mediates the relationship between job structure and self-efficacy.

## 5. Conclusions

In conclusion, organizations have often assumed that their most capable employees in the most complex roles would lead AI adoption. Our research suggests a fundamental, perhaps curious, paradox. The very complexity that makes AI seem most valuable may also make it least adoptable, while the simplicity that enables full automation can eliminate the human engagement necessary for efficacy development. Our findings suggest that the path to successful AI integration may lie not at the extremes but in the moderate complexity zone where human and artificial intelligence can develop a productive partnership through iterative interaction.

## Figures and Tables

**Table 1:** Definitions and Distinctions of Key Constructs.

Construct	Definition	Theoretical Foundation	Psychological/ Structural Focus	Distinguishing Characteristic
Job Complexity	The extent to which a job entails autonomy, non-routine tasks, decision latitude, and a range of skills required for effective task execution	Shalley et al. (2009); Morgeson and Humphrey (2006); Wood (1986)	Task structure—“What does my work demand?”	Structural job characteristic that shapes interaction opportunities with AI; determines task-technology alignment
AI Self-Efficacy	Employees’ beliefs in their capability to successfully use AI to accomplish work tasks	Bandura (1997); Compeau and Higgins (1995)	Capability beliefs—“Can I use AI effectively?”	Confidence developed through mastery experiences; shaped by job complexity
AI Adoption Readiness	The extent to which individuals are cognitively, affectively, and intentionally prepared to integrate AI beyond minimal requirements	Armenakis et al. (1993); Rafferty et al. (2013)	Preparedness for integration—“Am I ready to integrate AI?”	Active preparation through deliberate skill investment; bridges capability beliefs and action
AI Adoption Behavior	The frequency and extent of actual AI use in daily work activities	Venkatesh et al. (2003)	Actual usage—“How often do I use AI?”	Observable action; behavioral manifestation of self-efficacy and readiness

**Table 2:** Hypotheses and Theoretical Rationales.

Hypothesis	Theoretical Rationale
**H1**. Job complexity has an inverted-U shaped relationship with AI self-efficacy, such that AI self-efficacy initially increases as job complexity moves from low to moderate levels but decreases as job complexity increases beyond moderate levels to high complexity.	At low complexity, excessive automation limits human–AI interaction, preventing mastery experiences. At moderate complexity, alignment between task demands and AI capabilities fosters positive mastery experiences, boosting confidence. At high complexity, AI’s bounded capabilities create misfit, leading to failure experiences and diminished self-efficacy.
**H2**. AI self-efficacy is positively related to AI adoption readiness.	Employees with high AI self-efficacy (developed through mastery experiences) allocate cognitive and emotional resources toward learning and planning for AI use, fostering preparedness to adopt AI.
**H3**. AI adoption readiness is positively related to AI adoption behavior.	AI adoption readiness provides bridge between intention and action, equipping employees with procedural knowledge and confidence to translate readiness into consistent AI use.
**H4**. AI self-efficacy is positively related to AI adoption behavior.	AI self-efficacy influences persistence and resilience during AI use, helping employees overcome output variability and perceived failures, thereby sustaining continuous AI engagement.

**Table 3:** Descriptive Statistics and Zero-Order Correlations for Study 1 Variables.

Variable	M	SD	1	2	3	4	5	6	7	8
1. AI Self-Efficacy	2.49	1.07	0.90							
2. AI Readiness	2.70	1.18	0.70 ***	0.90						
3. AI Use	1.81	0.86	0.57 ***	0.66 ***	0.91					
4. Job Complexity	3.60	0.66	0.00	−0.02	0.09	—				
5. Learning Sources	1.78	0.94	0.27 ***	0.31 ***	0.37 ***	0.15 **	—			
6. Age	3.73	1.39	−0.09	−0.03	0.05	0.00	0.05	—		
7. Race	0.80	0.40	−0.06	0.02	0.02	0.03	0.06	0.02	—	
8. Academic Role	0.46	0.50	0.03	−0.04	0.19 ***	−0.03	0.18 **	0.21 ***	0.09	—
9. Tenure	9.26	8.32	−0.06	−0.06	0.05	0.18 **	0.07	0.51 ***	0.13 *	0.17 **

**Note.** *N* = 306. Cronbach’s alpha reliability coefficients are presented on the diagonal for multi-item scales. Age is an ordinal variable with categories representing age ranges (e.g., 18–25, 26–30, etc.). Race is coded 0 = non-White, 1 = White. Academic Role is coded 0 = non-faculty, 1 = faculty. Tenure is measured in years. * *p* < 0.05. ** *p* < 0.01. *** *p* < 0.001.

**Table 4:** Confirmatory Factor Analysis Model Fit Statistics for Study 1.

Model	χ^2^	df	*p*	SRMR	RMSEA	CFI	TLI	AIC	BIC	Δχ^2^	Δdf	*p*
Model 1: Four-factor model	100.72	30	<0.001	0.038	0.087	0.97	0.96	7055	7186	—	—	—
Model 2: Three-factor model	375.80	33	<0.001	0.053	0.182	0.85	0.80	7324	7444	275.08	3	<0.001
Model 3: Two-factor model	681.32	35	<0.001	0.084	0.243	0.73	0.65	7626	7738	580.60	5	<0.001

**Note.** *N* = 306. Model 1 represents the hypothesized four-factor model with items specified to load on their respective theoretical factors (AI Efficacy, AI Readiness, AI Use, and Job Complexity as distinct latent factors). Model 2 is a three-factor model with AI Efficacy and AI Readiness items loading on a single combined factor (AI Attributes), while AI Use and Job Complexity remain separate factors. Model 3 is a two-factor model with all AI items (Efficacy, Readiness, and Use) loading on a single factor, with Job Complexity as a separate factor. χ^2^ = chi-square; SRMR = standardized root mean square residual; RMSEA = root mean square error of approximation; CFI = comparative fit index; TLI = Tucker–Lewis index; AIC = Akaike information criterion; BIC = Bayesian information criterion. Δχ^2^ represents chi-square difference test compared to Model 1.

**Table 5:** Convergent and Discriminant Validity Analysis for Study 1 Measurement Model.

Construct	CR	AVE	MSV	ASV	1	2	3	4
1. Job Complexity	1.00	1.00	0.006	0.003	—			
2. AI Efficacy	0.90	0.76	0.51	0.30	−0.03	**0.87**		
3. AI Readiness	0.91	0.76	0.51	0.34	−0.03	0.72 ***	**0.87**	
4. AI Use	0.91	0.78	0.50	0.29	0.08	0.61 ***	0.71 ***	**0.88**

**Note.** *N* = 306. CR = composite reliability; AVE = average variance extracted; MSV = maximum shared variance; ASV = average shared variance. Bold values on the diagonal represent the square root of AVE. Values below the diagonal are inter-construct correlations from the CFA model. Job Complexity is measured with a single indicator, hence CR and AVE = 1.00. For adequate convergent validity: CR > 0.70, AVE > 0.50, and factor loadings > 0.50 (all factor loadings ranged from 0.74 to 1.00). For discriminant validity: (a) square root of AVE should exceed inter-construct correlations (Fornell-Larcker criterion), and (b) MSV and ASV should be less than AVE. All validity criteria were met. *** *p* < 0.001.

**Table 6:** Path Analysis Results in Study 1.

Predictors	AI Efficacy			AI Readiness			AI Use		
	**B**	**SE**	**β**	**B**	**SE**	**β**	**B**	**SE**	**β**
AI Efficacy	—	—	—	0.712 ***	0.063	0.699	0.198 *	0.078	0.211
AI Readiness	—	—	—	—	—	—	0.497 ***	0.073	0.537
Job Complexity (linear)	−0.108	0.089	−0.076	0.009	0.074	0.006	0.087	0.059	0.065
Job Complexity ^1^	−0.205 **	0.075	−0.167	0.143 *	0.068	0.115	−0.050	0.047	−0.043
Age	−0.035	0.051	−0.051	0.035	0.035	0.050	0.023	0.037	0.037
Race	−0.206	0.143	−0.088	0.213 *	0.103	0.089	−0.040	0.091	−0.018
Academic Role	0.023	0.116	0.012	−0.098	0.092	−0.051	0.253 **	0.085	0.143
Tenure	−0.001	0.008	−0.009	−0.007	0.006	−0.057	0.004	0.006	0.037
Learning Sources	0.271 ***	0.065	0.273	0.162 **	0.048	0.160	0.080	0.043	0.086
*R* ^2^	0.136			0.558			0.582		

**Note**. Unstandardized coefficients (B) and standardized coefficients (β) are presented with bootstrap standard errors based on 10,000 bootstrap samples. Job Complexity was mean-centered before creating the quadratic term. * *p* < 0.05. ** *p* < 0.01. *** *p* < 0.001. ^1^ Job complexity refers to the quadratic effect of job complexity.

**Table 7:** Descriptive Statistics and Zero-Order Correlations for Study 2 Variables.

Variable	M	SD	1	2	3	4	5	6	7	8
1. AI Self-Efficacy	3.24	1.28	0.93							
2. AI Readiness	3.56	1.36	0.84 ***	0.95						
3. AI Use	3.06	1.09	0.74 ***	0.74 ***	0.95					
4. Job Complexity	4.09	0.83	−0.06	−0.11	−0.02	0.89				
5. Learning Sources	2.58	0.99	0.30 ***	0.27 ***	0.30 ***	0.11	—			
6. Age	3.83	1.33	−0.08	−0.02	−0.03	0.12	−0.02	—		
7. Race	0.77	0.42	−0.06	−0.08	−0.10	0.08	0.07	0.02	—	
8. Academic Role	0.53	0.50	−0.26 ***	−0.28 ***	−0.13 *	0.33 ***	0.07	0.12	0.05	—
9. Tenure	8.92	8.11	−0.04	0.01	0.00	0.14 *	0.05	0.55 ***	0.10	0.13 *

**Note**. *N* = 246. Cronbach’s alpha reliability coefficients are presented on the diagonal for multi-item scales. Age is an ordinal variable with categories representing age ranges (e.g., 18–25, 26–30, etc.). Race is coded 0 = non-White, 1 = White. Academic Role is coded 0 = non-faculty, 1 = faculty. Tenure is measured in years. * *p* < 0.05. *** *p* < 0.001.

**Table 8:** Confirmatory Factor Analysis Model Fit Statistics for Study 2.

Model	χ^2^	df	*p*	SRMR	RMSEA	CFI	TLI	AIC	BIC	Δχ^2^	Δdf	*p*
Model 1: Four-factor model	107.90	48	<0.001	0.044	0.070	0.98	0.97	6551	6700	—	—	—
Model 2: Three-factor model	222.95	51	<0.001	0.047	0.116	0.95	0.93	6661	6798	115.05	3	<0.001
Model 3: Two-factor model	555.54	53	<0.001	0.064	0.194	0.84	0.80	6989	7120	447.64	5	<0.001

**Note**. *N* = 246. Model 1 represents the hypothesized four-factor model with items specified to load on their respective theoretical factors (Job Complexity, AI Efficacy, AI Readiness, and AI Use as distinct latent factors). Model 2 is a three-factor model with AI Efficacy and AI Readiness items loading on a single combined factor (AI Attributes), while Job Complexity and AI Use remain separate factors. Model 3 is a two-factor model with all AI items (Efficacy, Readiness, and Use) loading on a single factor, with Job Complexity as a separate factor. χ^2^ = chi-square; SRMR = standardized root mean square residual; RMSEA = root mean square error of approximation; CFI = comparative fit index; TLI = Tucker–Lewis index; AIC = Akaike information criterion; BIC = Bayesian information criterion. Δχ^2^ represents chi-square difference test compared to Model 1.

**Table 9:** Convergent and Discriminant Validity Analysis for Study 2 Measurement Model.

Construct	CR	AVE	MSV	ASV	1	2	3	4
1. Job Complexity	0.90	0.74	0.005	0.002	**0.86**			
2. AI Efficacy	0.93	0.82	0.79	0.47	−0.04	**0.91**		
3. AI Readiness	0.95	0.85	0.79	0.47	−0.07	0.89 ***	**0.92**	
4. AI Use	0.95	0.87	0.63	0.42	0.00	0.79 ***	0.79 ***	**0.93**

**Note**. *N* = 246. CR = composite reliability; AVE = average variance extracted; MSV = maximum shared variance; ASV = average shared variance. Bold values on the diagonal represent the square root of AVE. Values below the diagonal are inter-construct correlations from the CFA model. For adequate convergent validity: CR > 0.70, AVE > 0.50, and factor loadings > 0.50 (all factor loadings ranged from 0.76 to 0.95). For discriminant validity: (a) square root of AVE should exceed inter-construct correlations (Fornell-Larcker criterion), and (b) MSV and ASV should be less than AVE. All validity criteria were met. *** *p* < 0.001.

**Table 10:** Path Analysis Results in Study 2.

Predictors	AI Efficacy			AI Readiness			AI Use		
	**B**	**SE**	**β**	**B**	**SE**	**β**	**B**	**SE**	**β**
AI Efficacy	—	—	—	0.846 ***	0.041	0.794	0.344 ***	0.071	0.404
AI Readiness	—	—	—	—	—	—	0.340 ***	0.071	0.424
Job Complexity (linear)	−0.152	0.115	−0.100	−0.127	0.072	−0.078	0.085	0.064	0.065
Job Complexity ^1^	−0.204 *	0.089	−0.175	−0.059	0.054	−0.047	0.074	0.054	0.075
Age	−0.054	0.067	−0.056	0.038	0.042	0.037	0.006	0.040	0.007
Race	−0.218	0.172	−0.071	−0.112	0.113	−0.034	−0.125	0.115	−0.048
Academic Role	−0.654 ***	0.153	−0.256	−0.169	0.108	−0.062	0.152	0.099	0.070
Tenure	0.005	0.011	0.029	0.006	0.007	0.036	−0.001	0.006	−0.005
Learning Sources	0.424 ***	0.077	0.328	0.060	0.051	0.044	0.060	0.044	0.055
*R* ^2^	0.206			0.715			0.621		

**Note.** Unstandardized coefficients (*B*) and standardized coefficients (β) are presented with bootstrap standard errors based on 10,000 bootstrap samples. Job Complexity was mean-centered before creating the quadratic term. Em-dashes indicate paths not estimated in the model. * *p* < 0.05. *** *p* < 0.001. ^1^ Job complexity refers to the quadratic effect of job complexity.

## Data Availability

The data presented in this study are available upon request from the corresponding author due to the inclusion of confidential institutional information.
